# Biosafety for Dental Patients During Dentistry Care After COVID-19: A Review of the Literature

**DOI:** 10.1017/dmp.2020.252

**Published:** 2020-07-14

**Authors:** Adriana Abigail Siles-Garcia, Anais Gabriela Alzamora-Cepeda, Katherine Joselyn Atoche-Socola, Claudio Peña-Soto, Luis Ernesto Arriola-Guillén

**Affiliations:** School of Dentistry, Universidad Científica del Sur, Lima, Peru; Division of Oral Rehabilitation, School of Dentistry, Universidad Científica del Sur, Lima, Peru; Division of Periodontology, School of Dentistry, Universidad Científica del Sur, Lima, Peru; Division of Orthodontics, School of Dentistry, Universidad Científica del Sur, Lima, Peru

**Keywords:** Biosafety, patients, dentistry, COVID-19, SARS-CoV-2

## Abstract

The world is currently changing due to coronavirus disease 2019 (COVID-19), and the field of dentistry is no stranger to this. The care of patients in the dental office involves very strict biosafety protocols, and patients must be aware of the protection barriers implemented to allow satisfactory, safe dental care. The purpose of this study was to synthesize and analyze the management of the current biosafety standards for dental patients since the arrival of the COVID-19 pandemic. A bibliographic search of the main sources of information including MEDLINE (by means of PubMed), Scopus, Science Direct, SCIELO, and Google Scholar was carried out. Articles published without language restriction, systematic reviews, literature reviews, and observational studies were included. We identified the biosafety measures that must be taken before, during, and after dental practice following the arrival of COVID-19. The main measures include telephone triage, temperature taking on arrival at the office, the organization of the waiting room, washing hands before entering the office, knowing the auxiliary radiographic exams of choice and what type of treatment can be performed, albeit with restrictions. In conclusion, dental patients must comply with all the biosafety measures established by international protection standards and implemented by dentists before, during, and after dental practice to reduce the possibility of COVID-19 infection.

During the first 6 mo of 2020, the world has been exposed to a fearsome virus that has spread rapidly. This recent viral disease was named coronavirus disease 2019 (COVID-19) and is caused by a beta-coronavirus called severe acute respiratory syndrome coronavirus 2 (SARS-CoV-2).^1^ On December 31, the Wuhan Municipal Health Department (Hubei Province, China) notified the World Health Organization (WHO) of several cases of pneumonia of unknown origin, and due to the aggressive spread of the virus, a global pandemic was declared on March 11, 2020.^2^ The main routes of transmission of this virus are by close contact and by contact through droplets of the respiratory tract when an infected individual coughs, sneezes, or speaks.^3,4^ Various organizations at the international level have implemented a series of measures to stop the advance of COVID-19.^5-7^

Dentistry is one of the health professions most affected by this virus due to direct contact with the oral cavity of patients, because the dissemination of small droplets during sneezing or talking is the main route of transmission. Therefore, biosafety measures must be efficient to avoid possible cross-infection. The use of a mask is a very important protection barrier, especially the FFP2 masks with valves or the N95, with which 95% of aerial particles are filtered, being very helpful in an environment with high production of spray or splashes contaminated with saliva or blood.^8^ Likewise, recent research has demonstrated the efficacy of the use of mouthwashes before dental care, which could help decrease the amount of bacterial load by 68.4%.^9^

Patients in dental services are exposed to COVID-19 infection if dental professionals do not comply with the biosafety protection measures implemented by the COVID-19 regulations, which include the number and type of patients attended, facial barriers, body protection, disinfection of environments, and social distancing.^9^ It is important to point out that protection protocol measures should not only involve the personnel who provide dental care, but also the patients to reduce cross-contagion.^10,11^ Faulty control of patient protection can lead to contamination of the office environment, the personnel and even the patients themselves, further increasing contagion.^11^

In the event of any dental emergency, it is essential to acquire information through the medical history in which the patient must report the presence of any symptoms, such as respiratory distress, dry cough, fever, or odynophagia. In case of suspicion, the patient should be referred to hospital emergency services for testing, subsequent confirmation, and treatment if necessary.^12^ However, if the presence of the disease is ruled out, dental consultation can be carried out following the prevailing biosafety parameters. It is recommended to focus only on emergencies, acute pain, trauma, and infections of dental origin. Therefore, during dental care, patients require personal protective equipment (disposable shoe covers, cap, etc.), and protective equipment during clinical procedures should include the use of the rubber dam and high-power saliva suctioning as far as possible.^13^

With the declaration of lockdown, many dental treatments have been postponed, and professionals are only allowed to attend dental emergencies. However, after the lifting of the lockdown and care is restored, the “new normality” will require spacing of patient visits to meet biosafety measures, with protection barriers for the patient and disinfection of the dental environment after each procedure. Therefore, the purpose of this article was to describe the management of the latest biosafety standards for dental patients since the arrival of COVID-19, seeking to improve biosafety protocols and general protection during dental care.

## METHODS

The bibliographic search was performed using the main data sources from the international health science literature (MEDLINE) by means of PubMed, Scopus, ScienceDirect, SCIELO, and Google Scholar. The search was performed without language restriction from the source of the information until May 28, 2020. Systematic reviews and literature reviews were included in this research. Letters to the Editor, individual opinions, and books were excluded (Table 1).

**TABLE 1:** Questionnaire for Patients Before Dental Care

Questions
1) Do you have a fever or have you had a fever in the last 14 days?
2) Have you experienced a recent onset of respiratory problems, such as coughing or respiratory distress, in the last 14 days?
3) In the last 14 days, have you traveled to an already documented area of COVID-19?
4) Have you come into contact with a patient with confirmed COVID-19 infection in the last 14 days?
5) Have you come into contact with people who have had fever or documented respiratory problems in the last 14 days?
6) Have you had contact with many unknown people or have you recently participated in an event or meeting?

### Considerations to Be Taken Before Dental Care

On any suspicion of viral infection, dental appointments must be canceled and the patient should be advised to immediately go to the hospital. On the other hand, in the absence of symptoms and with the need for a dental appointment, a questionnaire will give by phone to rule out a possible infectious process, and if the patient is considered virus-free, an appointment will be scheduled (Table 1). Once in the office, the temperature of the patient is measured with a digital thermometer on the forehead to identify possible fever. The thermometer must be disinfected with 70% ethyl alcohol after each use as recommended by the WHO.^14^ If the patient is in an acute febrile state, dental care will be stopped, the appointment rescheduled, and the patient will be advised to go to the doctor.^14,15^

In the case of patients with a temperature below to 98.6°F, or 37.3°C, but with an affirmative answer on the questionnaire (Table 1), the treatment will be postponed to 14 days after the exposure event. The patient is instructed to initiate quarantine and report any fever or flu-like symptoms to the local health department.^15^ On the other hand, patients with a temperature above of 98.6°F, or 37.3°C, and an affirmative answer to the questionnaire will be considered as suspicious or at risk for COVID-19, and the appointment will be postponed, and the patient referred to emergency medical services for diagnosis.^15^

### Considerations for Patients to Take During the Dental Consultation

#### Use of Rinses

Many authors recommend the use of mouthwashes administered before dental care to help decrease the number of bacteria and/or viruses. However, some research has shown that the use of chlorhexidine is not effective for eliminating COVID-19. Nonetheless, there are 2 antiseptic options with oxidative content that favorably decreases the salivary load of the virus without causing damage to the oral mucosa, and these are: hydrogen peroxide diluted at 1%, povidone 0.2% or cetylpyridinium chloride (CPC) 0.05-0.1%.^15-17^ According to the studies available, the oral mouthwash of choice is hydrogen peroxide because COVID-19 is vulnerable to oxidation.^18^ To obtain 15 mL of rinse at a concentration of 1%, 5 mL of 10 volume hydrogen peroxide increasing to 10 mL of distilled water can be used.^17^

#### Aerosol Control

A recent study showed there are around 38 types of microorganisms in the air in a dental office, including *Legionella pneumophila*, the causative agent of severe pneumonia.^19-21^ On the other hand, several studies have shown that many dental procedures produce contaminated aerosols, and droplets remain in the environment for a considerable length of time.^15,22^ As described in several studies, COVID-19 is transmitted through the air when an infected individual coughs, laughs, sneezes, and speaks to a susceptible individual in close physical proximity. Therefore, disease propagation is the most important concern in clinics and hospitals, because it is difficult to avoid the generation of large amounts of droplets and aerosols mixed with the patient’s saliva and even blood during dental practice. Dental instruments, such as high-speed handpieces use aerosol oil to make the turbine rotate at high speed and run under running water.^15,23^ The particles generated are so small that they are able to remain in the air for a long period of time before depositing on environmental surfaces or in the respiratory tract. Therefore, COVID-19 has the potential to spread through aerosols from infected people.^15^

Several health institutions have recommended that rotating instruments be used as little as possible. In specific cases, chemical or mechanical techniques for caries removal can be considered. If the use of rotating instrumentation is really necessary, absolute isolation is recommended. Furthermore, ultrasonic instrumentation is effective in removing plaque and calculus deposits, and manual scaling and root planning is recommended, as both techniques achieve the same result.^21,24^ When lockdown has been lifted, the use of rotating instruments will be reincorporated, maintaining rigorous compliance with the established biosafety protocols.

### Hand Washing Before and After Dental Care

Hand washing with plenty of soap and water is an extremely important element for infection control for both patients and professionals alike.^25^ Several studies have confirmed that adequate hand washing can break the transmission cycle of respiratory diseases and reduce the risk of transmission by 6 to 14%.^24^ There have also been reports of fecal-oral transmission of COVID-19, further emphasizing the need for handwashing in dental practice. Although hand washing is a general requirement, compliance is relatively low, making infection control a great challenge during care.^15^

### Precautions to Consider During Dental Care

Currently, some researchers have considered pediatric populations as one of the most vulnerable to possible contagion by COVID-19. In addition to the transmission routes presented by this virus (inhalation of droplets, sneezing, direct contact, and saliva), additional risks in the care of pediatric patients involve the use of removable orthodontic devices and auxiliary elements. These devices induce a higher risk of contamination if biosafety protocols are not optimally followed. On the other hand, pediatric patients should always be accompanied by someone who will also be in close contact with the operator, increasing the risk of cross-contamination.

Due to the long incubation period of approximately 2 to 14 days, the signs and symptoms of infected pediatric patients may go unnoticed as the symptomatology may be mild.^26^ For this reason, both parents and infants should be considered potential carriers of COVID-19.

Upon arrival at the dental office, the child and the accompanying person should be asked to wash their hands and face, using alcohol gel for the hands. Preferably, disposable surgical footwear covers will be placed over the shoes. Before proceeding with the treatment, the previously mentioned mouthwash of choice will be used by cleaning with a gauze impregnated with the rinse to reduce the risk of ingestion.^27,28^

During the treatment, only the patient, operator, and assistant should be present, although if necessary, a maximum of 1 accompanying person will be allowed. The patient must be able to collaborate with the treatment being carried. Children unable to collaborate with the treatment should be referred for care under sedation or general anesthesia.^28^

At the end of the treatment, the air space will be cleaned with a disinfectant spray, waiting 2 min before opening the door. The patient and accompanying person will leave the unit and will be instructed to wash their hands and face before leaving the dental office.

On the other hand, the generation of greater quantity of fluids and droplets during oral surgery procedure leaves dentists exposed to possible contagions of diseases that could be transmitted to patients if adequate protection barriers are not implemented.^23,29-31^ It is important to emphasize that, in the absence of adequate safety elements, the health of both the patients and the work team cannot be guaranteed; therefore, dental procedures cannot be undertaken.^30^

Several studies have suggested that a minimum distance of 1-2 m must be maintained, with no bodily contact, such as kissing or hugging. It has also been suggested to have a container at the entrance of the office to disinfect footwear using a bleach solution of 1/5 of sodium hypochlorite in 4/5 of water, 800 mL of water + 200 mL of sodium hypochlorite remaining at 10,000 ppm, which should be changed every 4 h.^17,30^ All these considerations should be taken into account when preparing for a consultation to rule out any type of infection and decide whether to continue or cancel an appointment.

The recommendations state that patients should wash their hands with soap and water or antibacterial gel for a minimum time of 20 s. After that, documents requiring a signature, such as informed consent, medical records, and forms required by the insurers, can be handled.^21^ Electronic banking services should be used for payment because receiving bills or coins requires their placement in plastic bags and being sprayed with disinfectant. In addition, pens have also been described as possible vehicles of the spread of the virus^31^; therefore, each worker and patient should have their own pen for individual use.

Subsequently, before entering the consultation, the patient should perform a new hand wash and thereafter remain with their hands on their chest and not touch anything.^29^ The literature recommends patients washing their hands twice before entering the procedure and three times thereafter. The patient is instructed where to stay, and if the office must be left, the entire protocol should be repeated upon reentry if the use of washrooms should be avoided, and if necessary, the area should immediately be disinfected.^23,30^

On the other hand, extraoral radiographic examinations such as panoramic radiography or cone beam computed tomography (CT) are considered good alternatives to reduce contact with the patient saliva. Nevertheless, the cost of these auxiliary examinations for the patient and their requirement by the clinician must be considered.^17^

Before starting the procedure, the dentist should ensure that an anesthetic boost is not necessary. It is ideal to consider the use of truncal anesthesia, plus an infiltrative supplement. According to the surgical technique to be used in the area to be treated, the use of resorbable sutures is recommended to decrease session time. The area is irrigated with needles, and aspirated to reduce the amount of spray. Patients with trauma or maxillofacial infections generally present an elevated temperature. However, according to the epidemiological history, etiology, clinical examination, blood test, and chest CT scan, this differs from that of COVID-19.^16,29^

After the procedure, all disposable materials are removed from the patient and hand washing is requested. Recommendations for postoperative care and the prescription of medication are given to the patient,^21^ followed by another hand washing and exit from the dental office. When exiting, the patient should avoid touching office surfaces, and finally, hands should be washed with antibacterial gel.^31^

Ideally, care in these cases should be carried out in rooms with negative pressure, such as wards, reducing the number of staff present.^16,31^ Alternatively, patients should receive care in an isolated room with good ventilation. In life-threatening cases, the patient should be immediately admitted to a hospital and chest CT performed, if available, because reverse transcription polymerase chain reaction testing takes a long time.^32^

Dental emergencies are closely related to pain management, and endodontists are closely involved with the evaluation and treatment of odontogenic pain and inflammation. It is highly likely that dental practices can treat some patients with asymptomatic COVID-19 infections. A recent article described a set of recommendations for the management of dental emergencies, which concluded that the use of ibuprofen 600 mg with paracetamol 500 mg would be effective for symptomatic irreversible pulpitis, symptomatic apical periodontitis, acute apical abscess.^33-35^

## DISCUSSION

The aim of this research was to present the biosafety rules and protocols that patients should take into account if they require dental care after the appearance of COVID-19. With the active spread of this virus, patients should be treated according to rigorous biosafety parameters to avoid cross-contamination before and during dental care especially, since the full practice of dentistry has been reopened in different cities and because the dental urgencies and emergencies cannot be postponed in most of the cases.

Before consultation, all patients, symptomatic or otherwise, should answer a questionnaire, followed by temperature measurement to rule out any infectious process. This care protocol must be maintained because all patients are considered as possible carriers. If a patient presents fever, the appointment will be cancelled and postponed, and the patient will be referred to emergency medical services to definitive rule out possible infection. Patients should await care within a safe environment, strictly following biosafety protocols. In the waiting room, it is recommended to remove magazines, brochures, or any other means or surface through which the virus can be transmitted among the patients present in that space. Constant disinfection of the environment should be maintained, and patients should be seated at least 1 m away from each other in a ventilated environment. Patients should not be accompanied, but in the case of accompanying persons, they will remain outside until the end of the dental care.^14,15,35-37^

Studies have shown that the use of precare oral rinses decreases the bacterial and/or viral load. Although COVID-19 still remains with the application of mouthwashes, it has been found to be susceptible to oxidation. Therefore, based on the results of many studies, the mouthwash of choice is hydrogen peroxide, which has oxidative capacity while not causing damage to the oral mucosa.^15-18^ One aspect of controversy is the cytotoxicity that the mentioned rinse can present. However, a study comparing three types of mouthwashes indicated that diluted rinses, such as in this case, would not have any additional adverse effects even if used as for prophylactic purposes.^39^ In addition, to prevent the spread of dental aerosols (released particles less than 50 microns in diameter) that are produced from dental instruments, such as like ultrasonic scalers, air-water syringes, dental handpieces when using rotating systems, which are a source of emission of microorganisms and even with droplets that can be produced and could generate a risk of contagion during dental care, many authors suggest the use of chemical or mechanical techniques for the elimination of caries. If rotary instrumentation must be used, absolute isolation is indicated, and if scaling and root planning are necessary, it is recommended to use a manual technique.

All specialties follow their own procedures in dental care. Each patient must strictly comply with the biosafety regulations. This review suggests a protocol to follow from admission to the dental office, the procedure, and the end of treatment. In the area of pediatrics, the application of a mouthwash should be modified to avoid toxicity due to ingestion, and an accompanying person may be allowed provided this is really necessary. On the other hand, in oral surgery, great importance is given to hand washing; it is suggested to perform hand washing twice before and three times after the procedure. In addition, once admitted to the consultation, the hands of the patient must remain on their chest and not touch any object, because in the event of violating this rule or leaving the office, the entire protocol must be repeated upon re-entry. Dental emergencies are closely related to the area of endodontics and maxillofacial surgery, the treatments of which are all associated with pain management. For this, according to many studies, medication for primary management is essential, among which the combination of ibuprofen 600 mg + paracetamol 500 mg is of note.^26-31,33-35,39-41^

Although several studies have described the current parameters of care to be implemented after the appearance of COVID-19, established and organized protocols for dental consultation remain to be developed.^42,43^ It is recommended to standardize the biosafety standards in an orderly and sequential way to guide patients and provide knowledge regarding care in the dental office, with possible variations, depending on the characteristics of the patient and the treatment required. Nonetheless, the information provided in this study needs to be expanded as studies on biosafety standards during COVID-19 continue to be published.

## CONCLUSIONS

Taking into account the different conditions of exposure to COVID-19 among patients before dental care, patients must comply with all the precare standards that include regulatory questionnaires and physical examinations.

Dental patients must comply with the biosafety measures established by international protection standards and implemented by their dentists before, during, and after the consultation, because this compliance will decrease the possibility of COVID-19 transmission.
